# A Modification of Lister’s Antiseptic Dressing

**Published:** 1882-04

**Authors:** James L. Little

**Affiliations:** Professor of Clinical Surgery in the University of the City of New York; Professor of Surgery in the University of Vermont, etc.


### Abstracts.

A MODIFICATION OF LISTER'S ANTISEPTIC DRESSING.* By JAMES L. LITTLE, M.D., Professor of Clinical Surgery in the University of the City of New York; Professor of Surgery in the University of Vermont, etc.

Although having full confidence in Mr. Lister's antiseptic method, I, like many others, have long recognized the great difficulty that must needs be experienced by the general practitioner in attempting to carry out the minute details of the dressing, and have for a long time been hoping that a more simple method, equally efficacious, might be devised.

Dr. Markoe's "through drainage" was a decided step in this direction—antiseptic in character, simple in detail, and successful in result. This method, however, is appropriate only where drainage is necessary, but, simple and efficient as it is, it requires a certain degree of attention, which, while easy for the hospital surgeon, is not sufficiently so to guarantee its extended use by the physician in charge of a large general practice.

Furthermore, the materials necessary for fully applying Mr. Lister's dressing are somewhat expensive, a very important fact when we consider that the majority of accidents and operations that call for this procedure occur among those who are able to bear but little expense.

I have for several years been surgeon to a large factory in this city, in which three thousand hands are employed, and where injuries by machinery are quite frequent. These injuries consist chiefly of wounds of the hands and fingers, caused by their being

*Read before the New York Surgical Society, November 8, 1881.

caught in the cog-wheels and other parts of the machinery. In many cases the fingers are torn off, tendons are pulled from their sheaths, joints are opened, and the hands are often severely crushed and lacerated. In all of these cases I have, for the past six years, been using the following simple antiseptic dressing: Having put the parts in a condition for dressing, I wash the wound in a solution of carbolic acid of the strength of one to twenty; I then cover the parts with a thick layer of borated cotton, and then snugly and evenly apply a simple gauze bandage. At first I used bandages made of antiseptic gauze, but for the past three years have used those of plain uncarbolized cheese cloth. These thin bandages distribute the pressure more evenly over the cotton, and are more evenly saturated with fluids than those made of unbleached muslin.

The patient is instructed to keep the outside of the dressing wet with a solution of carbolic acid of the strength of one to one hnndred. I frequently employ Squibb's solution of impure carbolic acid, which is of the strength of one to fifty, and which, when mixed with an equal bulk of water, gives a solution of the desired strength.

The parts should be kept at rest, and the dressings may be left undisturbed for several days, unless there is pain, rise of temperature, or discharge through the dressings. These conditions are always to be considered indications for re-dressing.

In many cases where rubber drainage tubes have been used they may be removed at the second dressing, and, if catgut has been used for sutures, this second dressing can be allowed to remain on for an indefinite period. In a number of cases of lacerated wounds I have allowed the first dressing to remain on until the wound has entirely healed. In these cases the external use of carbolic lotion was discontinued after the fifth or sixth day, and the dressings would become dry and hard, the wound healing, as it were, "under a scab."

The patient should be instructed to loosen the bandage at once if any pain occurs.

My experience with this dressing covers, as I have said, a period of about six years, during which time I have treated nearly three hundred cases of open wounds. Not one of this number has been followed by inflammatory symptoms. Extensive lacerated wounds have healed, and dead tissue has sloughed away, without giving rise to any of the so-called symptoms of inflammation. Neither pain, redness, heat, swelling, nor constitutional disturbance has resulted. In no case has there been reddening of the lymphatics or tenderness of the glands. No counter openings have been necessary. Pain has been entirely absent, so that anodynes have not been needed, save in a single case, and that for one night only, to control slight restlessness.

These results are the more remarkable from the fact that many of these patients were in an unhealthy condition, some suffering from anæmia, some from cardiac disease, phthisis, and the like.

Recently I used this modified dressing in St. Vincent's Hospital, in a case of amputation of the leg. This case did as well as any case could have done under the most rigid Lister dressing.

The value of cotton-wool as an antiseptic dressing is, I think, not fully appreciated by the profession. M. Guérin, of Paris, in 1872, and since then Mr. Gamgee, of Birmingham, England, have called attention to its great value. Used in the way I have indicated, it seems to me to be as perfect an antiseptic dressing as the gauze and other materials recommended by Mr. Lister, while at the same time it is free from all objections that pertain to the latter, and which materially hinder their use by the general practitioner. If applied in sufficient quantities around an open wound, it protects it thoroughly from the "floating matter of the air" which is supposed to be the real inciter of suppuration. It is the best germ filter known to us. Tyndall, whose experiments were very carefully made, found that while filtering the air, and endeavoring to get it perfectly pure, atmospheric dust, which would readily pass through sulphuric acid and a strong solution of caustic potash, was completely stopped by ordinary cotton-wool.

I have used the very excellent borated cotton made by Mr. am Ende, of Hoboken, containing 15 per cent. of boraic acid.*

* A great deal of the so-called borated cotton sold by dealers is made with a solution of borax, instead of boracic acid, which can always be ascertained by burning a piece of the cotton; if the cotton has been properly prepared with boracic acid, the flame is of a bright-green color, but, if, as is generally the case, borax has been used, the flame will show very little of the green tint.

Keeping it wet externally with the solution of carbolic acid, in the manner already described, renders it more surely antiseptic.

To insure success in the cases where the dressing is used, full precautions as to rendering the instruments, sponges, and the hands of the surgeon aseptic, and the use of drainage tubes if necessary, should not be neglected. Catgut or torsion should be used to arrest hæmorrhage. The spray may be resorted to, if thought necessary. At the second dressing, I now usually apply carbolized oil, of the strength of one to twelve, to the wound to facilitate the removal of the cotton, which is otherwise apt to adhere after the first dressing.

I would state, in conclusion, that my experience, thus far, seems to show that this simple dressing, so easy of application, is as thoroughly antiseptic as Mr. Lister's appliances, and that it has the very decided advantage of doing away with the necessity for using costly "protective oil-silk," "Macintosh cloth," "carbolized gauze," etc., and give us a dressing that can be used by any one under any circumstances, be it in the city or in the country. The borated cotton is easily kept for months unchanged. The fact that the dressing need not be done oftener than once in several days will especially commend it to the country physician.

The success of this procedure in the treatment of large wounds after accident or amputation will increase its importance, and materially extend its field of usefulness.

